# RET expression and detection of *KIF5B/RET* gene rearrangements in Japanese lung cancer

**DOI:** 10.1002/cam4.13

**Published:** 2012-07-12

**Authors:** Hidefumi Sasaki, Shigeki Shimizu, Yoichi Tani, Masahiko Maekawa, Katsuhiro Okuda, Keisuke Yokota, Masayuki Shitara, Yu Hikosaka, Satoru Moriyama, Motoki Yano, Yoshitaka Fujii

**Affiliations:** 1 Department of Oncology, Immunology, and Surgery, Nagoya City University Graduate School of Medical SciencesNagoya, Japan; 2 Department of Pathology, National Hospital Organization, Kinki-Chuo Chest Medical CenterSakai, Japan; 3 Dako Japan IncTokyo, Japan; 4 GSP Research IncKawasaki, Kanagawa, Japan

**Keywords:** FISH, *KIF5B/RET*, lung cancer, RET expression

## Abstract

RET encodes the tyrosine kinase receptor of growth factors belonging to the glial-derived neurotrophic factor family. Recently, *RET* gene rearrangements with N-terminal of *KIF5B* gene were identified in lung adenocarcinomas from large-scale sequencing. We investigated *RET* mRNA expression by real-time reverse-transcriptase polymerase chain reaction (RT-PCR) assay using LightCycler, and *KIF5B*/*RET* gene rearrangements using newly established fluorescence in situ hybridization (FISH) analysis in surgically treated nonsmall cell lung cancer (NSCLC) cases. RET protein expression was also investigated by immunohistochemistry (IHC). This study included 157 surgically removed NSCLC cases for mRNA level analyses. The *RET/β* actin mRNA levels were not significantly different between lung cancer (6.359 ± 15.268) and adjacent normal lung tissues (8.205 ± 28.931, *P* = 0.6332). Tumor/normal (T/N) ratio of *RET/β* actin mRNA levels was not different within gender, stage, smoking status, and pathological subtypes. T/N ratio of *RET/β* actin mRNA levels was significantly higher in *KIF5B/RET* rearrangement samples (161.763 ± 123.488) than in wild-type samples (5.9013 ± 17.148, *P* = 0.044). Although RET IHC positivity was not perfectly correlated with *KIF5B/RET* arrangement, we have detected the *KIF5B/RET* rearrangements using FISH analysis. Thus, we have successfully introduced FISH for diagnosing *KIF5B/RET* positive lung adenocarcinoma. This method facilitates the molecular evaluation for *RET* fusions and could be applicable in clinical practice to detect lung cancer that may be responsive to RET inhibitors.

## Introduction

Lung cancer is a major cause of death from malignant diseases due to its high incidence, malignant behavior, and lack of major advancements in treatment strategy [1]. A great deal of progress has been made in the target therapy for nonsmall cell lung cancer (NSCLC), largely owing to the development of small-molecular inhibitors, such as epidermal growth factor receptor (EGFR) [2–6] and ALK [7]. The recent discovery of a fusion gene that joins the *KIF5B* and *RET* oncogene from large-scale sequencing [8, 9] in a subset of NSCLCs have added a novel molecular subtype to the classification scheme for adenocarcinomas. The importance of recognizing this molecular subtype was highlighted by an inhibition using RET inhibitor for *KIF5B/RET* overexpression cells [9]. Therefore, an accurate and practical assay is urgently needed to detect this molecular subset of lung cancer [9]. Currently, the methods available for detecting *EML4/ALK* rearrangement are reverse-transcriptase polymerase chain reaction (RT-PCR) and fluorescence in situ hybridization (FISH) [7, 10]. RT-PCR is a single detect test to detect the gene rearrangements; however, it generally requires good quality RNA and a multiplex system [11]. Thus, *EML4/ALK* status, at least that determined using FISH, does seem to be extremely important, and gold standard as a specific treatment sensitivity marker with respect to small-molecule inhibitors of ALK [12–14].

Our group has already screened the *KIF5B/RET* gene status using RT-PCR assay (Yokota et al., unpubl. ms.). In this study, we have investigated *RET* mRNA expression by real-time PCR using LightCycler (Roche Molecular Biochemicals, Mannheim, Germany), protein expression by immunohistochemistry (IHC) and *KIF5B/RET* gene rearrangement status using newly established FISH analysis in surgically treated NSCLC cases. The findings were compared with the clinicopathologic features and *KIF5B*/*RET* gene status.

## Material and Methods

### Patient samples

The study group included NSCLC patients who had undergone surgery at the Department of Surgery, Nagoya City University Hospital. All tumor samples were immediately frozen and stored at –80°C until assayed. Because Lipson et al. [9] demonstrated that the *KIF5B/RET* rearrangements were found within adenocarcinoma histology of NSCLC, we mainly focused on adenocarcinomas without *EGFR* mutations. The clinical and pathological characteristics of the 157 NSCLC patients for *RET* mRNA gene analyses were as follows: 104 (66.2%) were male and 53 were female; 127 (80.9%) were diagnosed as adenocarcinomas and 25 were diagnosed as squamous cell carcinoma; 105 (66.9%) were smoker and 52 were nonsmoker; and 105 (66.9%) were pathological stage I. *KIF5B/RET* rearrangements statuses were already investigated.

### PCR assay for *RET* gene

Total RNA was extracted from NSCLC and adjacent normal lung tissues using Isogen kit (Nippon gene, Tokyo, Japan) according to the manufactures' instructions. RNA concentration was determined using Nano Drop ND-1000 Spectrophotometer (Nano Drop Technologies Inc., Rockland, DE). About 10 cases were excluded for each assay because tumor cells were too few to sufficiently extract tumor RNA. RNA (1 μg) was reverse transcribed using First strand cDNA synthesis kit with 0.5 μg oligo (dT)_16_ (Roche Diagnostics GmbH, Mannheim, Germany) according to the manufactures' instructions. The reaction mixture was incubated at 25°C for 15 min, 42°C for 60 min, 99°C for 5 min, and then at 4°C for 5 min. The complementary DNA (cDNA) concentration was determined using Nano Drop ND-1000 Spectrophotometer. About 200 ng of each cDNA was used for PCR analysis. To ensure the fidelity of mRNA extraction and reverse transcription, all samples were subjected to PCR amplification with *β* actin primers kit (Nihon Gene Laboratory, Miyagi, Japan) using LightCycler FastStart DNA Master HybProbe Kit (Roche Diagnostics GmbH). The *RET* PCR assay reactions were performed using LightCycler FastStart DNA Master SYBR Green I kit (Roche Diagnostics GmbH) in a 20-μL reaction volume. The primer sequences for *RET* gene at kinase domain were as follows: the forward primer, 5′-ACAGGGGATGCAGTATCTGG-3′ (at exon 14) and the reverse primer, 5′-CCTGGCTCCTCTTCACGTAG-3′ (at exon 16). The cycling conditions were as follows: initial denaturation at 95°C for 10 min, followed by 40 cycles at 95°C for 10 sec, 61°C for 10 sec, and 72°C for 7 sec.

### RET IHC

Seventy-two cases of NSCLC were immunostained by automated methods (Dako Japan Inc., Tokyo, Japan) for C-terminal of RET expression using the ready-to-use mouse monoclonal RET Oncoprotein clone 3F8 (Vector Laboratories, Burlingame, CA) and 78 for the rabbit monoclonal RET antibody (EPR2871, 1:250) (Epitomics, Inc., Burlingame, CA) with Dako linker kit using intercalated antibody-enhanced polymer (iAEP) method [15]. Slides were scored for cytoplasmic staining intensity and distribution. An IHC score was assigned to each case according to the following criteria showing the designated staining pattern: 3+, intense, granular cytoplasmic staining; 2+, moderately, smooth cytoplasmic staining; 1+, faint cytoplasmic staining; and no staining. The extent of the staining area was determined as 0–10%, 10–25%, 25–50%, 50–75%, 75–90%, and 90%. Cytoplasm was stained either granular (G1) or diffuse (G2).

### RET FISH testing

Unstained 5-μm sections of formalin-fixed paraffin embedded tumor tissue were submitted to dual-color FISH analysis using four probe sets. The *KIF5B/RET* probe sets were developed at GSP Research Inc. (Kawasaki, Japan), labeled with TexRed and the FITC. Probe sets were as follows: set 1, 5′RET (490kb; around 43MB∼43.5MB)-TexRed and 3′RET (630kb; around 43.6MB∼44.2MB)-FITC at 10q11.2; set 2, 5′KIF5B (620kb)-TexRed and 3′RET (630kb)-FITC. FISH detection kit (K5599) was used (Dako Japan Inc.). Briefly, NSCLC slides were deparaffinized and then preincubated with K5599 vial 1 95∼99°C for 10 min. Five to eight drops of Protease digestion buffer at room temperature for 5∼15 min and then wash and dry up for the slides. Labeled probe sets (10 mL) were cohybridized at 37°C for 72 h after denaturing at 82°C for 5 min. Stringency wash was conducted at 65°C with saline-sodium citrate (SSC) for 10 min. Slides were counter stained with 4′6-diamidino-2-phenylindole (DAPI).

### Statistical analysis

Statistical analyses were carried out using the Mann–Whitney *U*-test for unpaired samples and Wilcoxon's singed rank test for paired samples. Linear relationships between variables were determined by means of simple linear regression. Correlation coefficients were determined by rank correlation using Spearman's test and χ^2^ test. The overall survival of lung cancer patients was examined using the Kaplan–Meier methods, and differences were examined using the Log-rank test. All analysis was done using the Stat-View software package (Abacus Concepts Inc. Berkeley, CA), and was considered significant when the *P*-value was less than 0.05.

## Results

### *KIF5B*/*RET* gene alteration and RET mRNA status in Japanese lung cancer patients

We have sequenced for arrangement status of *KIF5B/RET* gene for 371 NSCLC samples (Yokota et al., unpubl. ms.). Briefly, of 371 patients, from direct sequencing using cDNA samples, we found three translocation cases. We have included these cases and investigated further studies. The *RET/β* actin mRNA levels were not significantly different between lung cancer (6.359 ± 15.268) and adjacent normal lung tissues (8.205 ± 28.931, *P* = 0.6332; *n* = 157). Tumor/normal (T/N) ratio of *RET/β* actin mRNA level was not correlated with gender (male vs. female, *P* = 0.7636), age (age ≤65 vs. >65, *P* = 0.3204), and smoking status (smoker vs. nonsmoker, *P* = 0.6693). The T/N ratio of *RET/β* actin mRNA level was not correlated with pathological stages, lymph node metastasis, tumor invasion statuses, and pathological subtypes (adeno-carcinoma vs. others, *P* = 0.2652). The T/N ratio of *RET/ β* actin mRNA level was more than 20 in seven cases including three translocation cases. The T/N ratio of *RET/ β* actin mRNA level was significantly higher in *KIF5B/RET* fusion cases (161.76 ± 123.488) when compared with the wild-type cases (5.901 ± 17.148, *P* = 0.044) (Table 1).

**Table 1:** Clinicopathological data of 157 lung cancer patients

	RET		
	
Factors	No. of patients (*N* = 157)	T/N ratio of *RET/β-*actin mRNA levels	*P*-value
Mean age (years)	66.7 ± 9.0		
Stage			
I	105 (66.9%)	8.534 ± 30.678	NS
II	23 (14.6%)	6.981 ± 12.707	
III–IV	29 (18.5%)	11.603 ± 40.194	
Tumor status			
T1	73 (46.5%)	11.044 ± 39.731	NS
T2	62 (39.5%)	7.242 ± 21.568	
T3	7 (4.5%)	10.454 ± 16.874	
T4	15 (9.6%)	4.318 ± 13.079	
Lymph node metastasis			
N0	116 (73.9%)	8.587 ± 29.636	NS
N1	23 (14.6%)	5.414 ± 10.982	
N2	18 (11.5%)	15.690 ± 49.890	
Pathological subtype			
Adenocarcinoma	127 (80.9%)	10.465 ± 33.907	0.2652
Others	30 (19.1%)	2.137 ± 3.865	
*KIF5B/RET* rearrangement			
Positive	3 (1.9%)	161.76 ± 123.488	0.044
Negative	154 (91.8%)	5.901 ± 17.148	
Smoking			
BI = 0	52 (33.1%)	14.047 ± 45.428	0.6693
BI > 0	105 (66.9%)	6.312 ± 19.525	
Age			
65≤	80 (51.0%)	10.318 ± 3.579	0.9147
>65	77 (49.0%)	7.385 ± 3.350	
Gender			
Male	104 (66.2%)	6.473 ± 19.581	0.7636
Female	53 (33.8%)	13.584 ± 45.087	

T/N, tumor/normal; NS, not significant; BI, Brinkman index.

### IHC for C-terminal of RET

**Figure 1:**
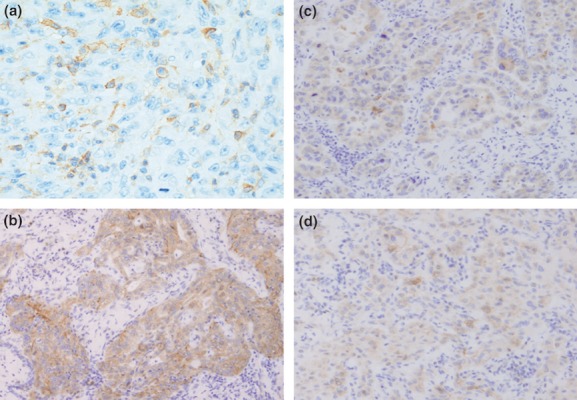
Using the 3F8 antibody, *KIF5B/RET* rearrangement case was weakly positively stained (1+) for RET protein (a). Using the rabbit monoclonal RET antibody, the staining intensity was 3+ (b), 2+ (c), and 1+ (d) for each *KIF5B/RET* fusion cases.

Using the 3F8 antibody, three cases in the *KIF5B/RET* positive group were weakly positively stained (1+) for RET protein (Fig. 1a). The staining intensity was weak for each case. The staining pattern was less than 10% and cytoplasmic. We have additionally performed IHC for *EGFR*-*Kras* wild-type and *KIF5B/RET* wild-type patients. The staining intensity was strong (3+) in six cases, moderate (2+) in 12 cases, and weak (1+) in 28 cases. Other cases showed almost no stained for RET. Using the rabbit monoclonal RET antibody, the staining intensity was 3+ (90%, G2), 2+ (90%, G2), and 1+ (50–75%, G2) (Fig. 1b–d) for each *KIF5B/RET* fusion cases, respectively. On the other hand, in 75 wild-type cases, four were 3+ (two were 50–75%, G1, one was 50–75%, G2, and one was 90%, G1), 13 were 2+ (two were 75–90%, five were 50–75%, two were 25–50%, three were 10–25%, and one was 0–10%), 43 were 1+ (14 were 0–10%, 16 were 10–25%, seven were 25–50%, three were 50–75%, and three were 75–90%), and 14 cases did not stained at all. Sixteen were G1 and 44 were G2. As for increased *RET* mRNA cases without translocations, one had only nuclear staining (1+, 75–90%), one had predominantly membranous staining (3+, 50–75%), one had 2+ (50–75%, G2), and one had 1+ (0–10%, G1) cytoplasmic staining.

### Cytogenic patterns of *KIF5B*/*RET* in FISH

**Figure 2:**
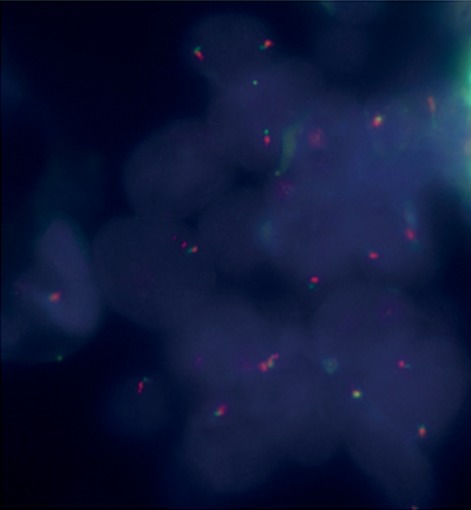
The typical signals for set 1 probes classified as positive for *RET* rearrangement, showing the normal signal (fused 3′ 5′ RET), the split/single 3′ RET, and the split/single 5′ RET signal.

**Figure 3:**
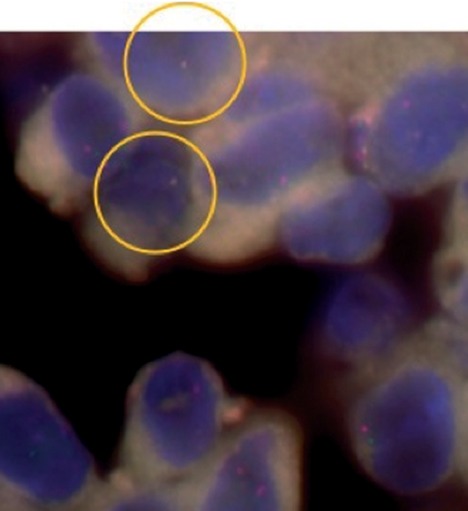
The typical signals for set 2 probes classified as positive for *KIF5B/RET* rearrangement, showing the normal signal (split red 5′-*KIF5B* and green 3′-*RET* signals), whereas if it was fusion, the signals showed only yellow signal per cells.

Figure 2 showed the typical signals for set 1 probes classified as positive for *RET* rearrangement, showing the normal signal (fused 3′ 5′ RET), the split/single 3′ RET, and the split/single 5′ RET signal. Normal signals are frequently seen in lung cancer cells that also display *RET* gene rearrangements. The typical *KIF5B/RET* fusion case should show distinct red and green signals separated within the same cell. To compile the frequency of positive cells for *RET* rearrangements, the 15% of cells carrying split red and green and single red (RET 5′ region) signals was considered as positive. Figure 3 showed the typical signals for set 2 probes classified as positive for *KIF5B/RET* rearrangement, showing the normal signal (split red 5′-*KIF5B* and green 3′-*RET* signals), whereas if it was fusion, the signals showed only yellow signal per cells. Two of three *KIF5B/RET* cases showed positive signals for FISH analyses using two probe sets, whereas wild case showed negative signals.

## Discussion

In this study, we have introduced newly developed methods for the detection of *KIF5B/RET* rearrangement using FISH, and the results showed a concordance between RT-PCR assay and FISH. We also investigated RET expression by IHC using two antibodies for C-terminal of RET; however, those did not correspond well part with the gene arrangement results evaluated with FISH. Although RET protein overexpression was not perfectly matched with the RET rearrangements, FISH analysis might be one of the candidate method for detecting *RET* gene status in lung cancer.

The *KIF5B/RET* fusion gene contains the entire kinase domain of RET and resulted in increased RET kinase activity. Oncogenic *PTC/RET* translocations detected in thyroid cancer and cell lines are sensitive in vitro and in vivo to RET inhibitors [16–19], suggested the possibility that sunitinib, sorafinib, vandetanib, or other RET inhibitors might also be clinically effective in *KIF5B/RET* lung cancers. Ba/F3 cells that were introduced with the full length *KIF5B-RET* gene (variant 1) showed interleukin 3 (IL-3) independent growth consistent with oncogenic transformation [9]. As cells with *KIF5B/RET* fusion gene overexpress the chimeric RET receptor tyrosine kinase and show spontaneous cellular transformation [20], inhibiting RET receptor tyrosine kinase may suppress tumor progression of *KIF5B-RET* fusion-positive lung cancer [20, 21]. The *KIF5B/RET* Ba/F3 cells were sensitive to sunitinib, sorafenib, and vandetanib, multitargeted kinase inhibitors that inhibit *RET*, but not to gefitinib, an EGFR kinase inhibitor [9]. Sunitinib, but not gefitinib, inhibited RET phosphorylation in the *KIF5B-RET* Ba/F3 cells [9].

**Figure 4:**
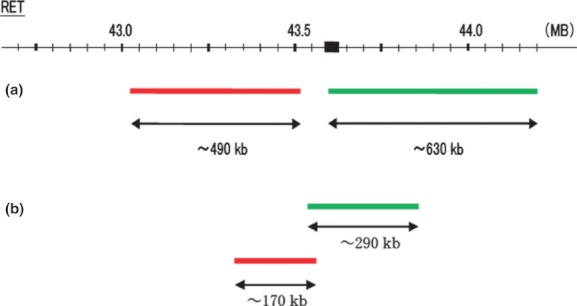
RET split probes. (a) 5′ (∼490kb) and 3′ (∼630kb) probes for *RET* gene (set 1). (b) 5′ (∼170kb) and 3′ (∼290kb) possible probes using BAC clones from National Center for Biotechnology Information [21].

**Figure 5:**
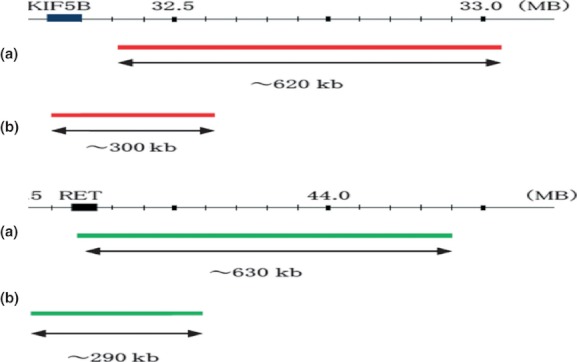
KIF5B/RET fusion probes. (a) 5′ KIF5B (∼620kb) and 3′ RET (∼630kb) probes for fusion gene (set 2). (b) 5′ KIF5B (∼300kb) and 3′ RET (∼290kb) possible probes using BAC clones from National Center for Biotechnology Information [21].

The recent study has been reporting the novel *KIF5B/RET* gene rearrangement found to occur in 2% (9/405) of Asian lung adenocarcinoma patients screened [9]. In our previous screen (Yokota et al., unpubl. ms.), we could only confirm the existence of 3/270 (1.1%) the *KIF5B/RET* rearrangements in lung adenocarcinomas. Several explanations exist for this discrepancy. Lung cancer encompasses a broad range of clinical subtypes, and the makeup of the two cohorts differed. Next, a racial difference between the studies about mutant *KIF5B/RET* may be existed, like *EGFR* gene mutations [2–6]. Because the arrangements were originally identified from massively parallel sequencing to generate comprehensive genomic profiles, we cannot conclude that differences were owing to our methodology as our PCR and sequencing methods. The technique used by Lipson et al. to identify *RET* arrangement was RT-PCR [8, 9]. This assay requires extraction of RNA from clinical samples, is not routinely performed in many diagnostic pathology laboratories, and suffers from some important limitations. Each PCR assay is *RET* fusion specific, and although assays may be multiplexed [11], they may not detect all possible *KIF5B/RET* gene arrangements, especially those involving novel fusion partners [15], a consequence of which may be false-negative results. Identification of a small fraction of lung cancers patients responsive to a molecular target therapy may have large clinical impact as highlighted by the recent FDA approval of crizotinib for ALK-rearrangement lung cancers [13]. This was based on the results of clinical trials for advanced lung cancers with *ALK* rearrangements, and early responses in *ALK* rearrangement assessed by FISH [13, 14]. Accurate and rapid screening of rearrangement is important in lung cancers, especially in determining their eligibility for molecular target therapy. Thus, we forward to step FISH analysis, which is gold standard for detection of *EML4/ALK* rearrangement. FISH enables the pathologist to perform a more reliable quantification of the genomic alteration. Takeuchi et al. [21] had undergone *KIF5B/RET* FISH analyses using BAC clones from National Center for Biotechnology Information. However, the length of each probe might be between 160 and 300 kb. In our analyses, we mixed 3–5 BAC clones from GSP Research Inc. (Figs. 4 and 5). Thus, the length of each probes for our *KIF5B/RET* analyses were between 490 and 630 kb. This may improve the specificity and sensitivity for *KIF5B/RET* rearrangements detection. In our preliminary experiments, the increase in the probe length increased the brightness of our FISH results.

However, even if we would like to expand the study, because the availability of tumor samples in the cohort was limited, we were unable to perform FISH in most patients. Most patients with advanced stage of NSCLC had only small tissue samples and the samples were mostly used for clinical diagnosis, leaving limited residual samples for molecular diagnosis. FISH methods need specific sample handling, such as tissue processing and method of fixation, standardization of reagents and protocols, standardized reporting criteria, and implementation of quality assurance procedures, including the requirement for laboratory accreditation and external proficiency assessment [22]. Paik et al. [23] showed that *EML4/ALK* rearrangement detection using IHC staining correlated well with FISH. IHC staining could be applied broadly in routine biopsy tissue diagnosis in clinical practice and thus we performed IHC using commercially available antibody and iAEP methods. Yang et al. [24] expand on previous experience [25] by evaluating the approach of initial testing IHC with the commercially available monoclonal ALK antibody and an enhanced detection system paired with subsequent FISH testing. The report suggested that IHC test was sensitive but not specific [24]. Similar strategies have been proposed using another commercially available antibody [26]. Thus, development and validation of strategies to improve effective identification of the patient population with strategies incorporating IHC or other techniques are important and likely to assume a place in clinical practice. Diffuse cytoplasmic staining (G2), more than 50% using EPR2871 might be correlated with *RET* translocation. There was a case where there was an elevated *RET* mRNA and protein expression, but no detectable rearrangement. This would suggest the presence of a novel translocation. Prospective study is needed to compare the usage of RT-PCR, FISH, and iAEP IHC. We only validated the result of RT-PCR using FISH or iAEP IHC using commercially available antibody in a limited number of patients.
